# Isothermal Technologies for HPV Detection: Current Trends and Future Perspectives

**DOI:** 10.3390/pathogens13080653

**Published:** 2024-08-02

**Authors:** Elda A. Flores-Contreras, Everardo González-González, Gerardo de Jesús Trujillo-Rodríguez, Iram P. Rodríguez-Sánchez, Jesús Ancer-Rodríguez, Antonio Alí Pérez-Maya, Salomon Alvarez-Cuevas, Margarita L. Martinez-Fierro, Iván A. Marino-Martínez, Idalia Garza-Veloz

**Affiliations:** 1 Departamento de Patología, Facultad de Medicina, Universidad Autónoma de Nuevo León, Francisco I. Madero y Dr. E. Aguirre Pequeño s/n, Mitras Centro, Monterrey 64460, Mexico; elda.florescn@uanl.edu.mx (E.A.F.-C.); jesus.ancer@uanl.mx (J.A.-R.); salomon.alvarezcv@uanl.edu.mx (S.A.-C.); 2 Molecular Medicine Laboratory, Unidad Académica de Medicina Humana y Ciencias de la Salud, Universidad Autónoma de Zacatecas, Zacatecas 98160, Mexico; dnarnaprot@gmail.com (E.G.-G.); entogerry36@gmail.com (G.d.J.T.-R.); margaritamf@uaz.edu.mx (M.L.M.-F.); 3 Laboratorio de Fisiología Molecular y Estructural, Facultad de Ciencias Biológicas, Universidad Autónoma de Nuevo León, San Nicolás de los Garza 66455, Mexico; iram.rodriguezsa@uanl.edu.mx; 4 Departamento de Bioquímica y Medicina Molecular, Facultad de Medicina, Universidad Autónoma de Nuevo León, Francisco I. Madero y Dr. E. Aguirre Pequeño s/n, Mitras Centro, Monterrey 64460, Mexico; antonio.perezmy@uanl.edu.mx

**Keywords:** HPV, NAATs, INAATs, isothermal amplification, cancer detection

## Abstract

The human papillomavirus (HPV) is a non-enveloped DNA virus transmitted through skin-to-skin contact that infects epithelial and mucosal tissue. It has over 200 known genotypes, classified by their pathogenicity as high-risk and low-risk categories. High-risk HPV genotypes are associated with the development of different types of cancers, including cervical cancer, which is a leading cause of mortality in women. In clinical practice and the market, the principal tests used to detect HPV are based on cytology, hybrid detection, and qPCR. However, these methodologies may not be ideal for the required timely diagnosis. Tests have been developed based on isothermal nucleic acid amplification tests (INAATs) as alternatives. These tests offer multiple advantages over the qPCR, such as not requiring specialized laboratories, highly trained personnel, or expensive equipment like thermocyclers. This review analyzes the different INAATs applied for the detection of HPV, considering the specific characteristics of each test, including the HPV genotypes, gene target, the limit of detection (LOD), detection methods, and detection time. Additionally, we discuss the tests available on the market that are approved by the Food and Drug Administration (FDA). Finally, we address the challenges and potential solutions for the large-scale implementation of INAATs, particularly in rural or underserved areas.

## 1. Introduction

Human papillomavirus (HPV) is a double-stranded DNA (dsDNA) virus belonging to the family *Papillomaviridae*, characterized by being naked viruses and having a wide range of hosts (Mammals, reptiles, birds, and fish) [1]. Currently, more than 200 HPV genotypes have been identified, which have different tissue specificity and infection rates [2]. Structurally, the HPV comprises a capsid comprising 72 molecules of the L1 protein and ~12 molecules of the L2 protein [1].

HPV spreads through direct skin-to-skin contact and is the most prevalent sexually transmitted infection affecting the reproductive tract, infecting cutaneous and mucosal epithelial tissue cells [3,4].

About 90% of HPV infections do not cause symptoms since they are eliminated by the immune system or are inactivated within the first- or second-year post-infection [5].

Nevertheless, the remaining 10% of HPV infections cause lesions that give rise to malignant neoplasms, developing cancer and being responsible for 5% of all cancers worldwide.

HPV-related cancers can develop in multiple tissues, such as the cervix, penis, anus, vulva, vagina, and oropharynx [6,7]. Notably, the virus can be in two forms: episomal (extrachromosomal elements) and integrated into the host’s genome. Integration into the host genome constitutes a significant molecular event in the process of carcinogenesis [8,9,10,11,12].

The International Agency for Research on Cancer classifies HPV according to its pathogenicity as high-risk and low risk [7,13]. Among the high-risk HPV genotypes are 16, 18, 31, 33, 35, 39, 45, 51, 52, 56, 58, 59, 66, and 68. Genotypes 16 and 18 are the most predominant types and are responsible for more than 70% of cervical cancers [7,13]. Low-risk genotypes include types 6 and 11, which are not carcinogenic, but they cause warts (cutaneous and anogenital) and mild dysplasia (cervix) [7,13,14].

HPV has also been classified based on variations in the L1 gene sequence (viruses must have a greater similarity than 60% to be of the same genus), grouping into five phylogenetic genera: *Alphapapillomavirus* (Alpha-HPV), *Betapapillomavirus* (Beta-HPV), *Gammapapillomavirus* (Gamma-HPV), *Mupapillomavirus* (mu-HPV), and *Nupapillomavirus* (nu-HPV) [15,16]. Most existing HPV genotypes correspond to the Alpha-HPV, Beta-HPV, and Gamma-HPV [15,16]. Regarding the Alpha-HPV genus, it is made up of high-risk and low-risk carcinogenic genotypes. The beta-HPV type resides in the hair follicles on the back of the hand, forehead, and genital skin. Furthermore, the beta-HPV and gamma-HPV genera are found in non-cutaneous anatomical sites such as the anal canal, nasal mucosa, and oral cavity [16,17,18].

**Figure 1:**
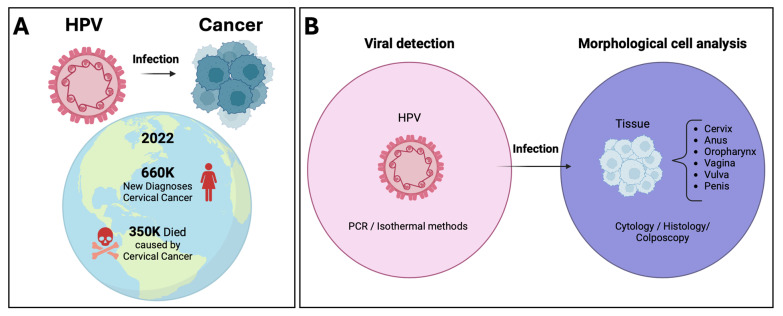
A summary of the global health problem caused by HPV and the methodologies used for its detection for subsequent management. (**A**) HPV is responsible for causing different types of cancer in the world population, with cervical cancer being the most prevalent. In 2022, it is estimated that 600,000 new cases of cervical cancer were diagnosed globally, and approximately 350,000 people died because of cervical cancer. (**B**) There are two main approaches to addressing the cancer problem caused by HPV. The first is the direct detection of HPV, and the second is the detection of the effects of HPV infection on cells or tissues.

The HPV-16 and HPV-18 genotypes belong to the Alpha-HPV genus and are classified as high-risk; they are the main ones responsible for the development of cervical cancer, representing 60 to 90% of cases, in addition to being one of the leading causes of mortality in women [16,19]. In 2022, around 660,000 women were diagnosed with cervical cancer worldwide, and it is estimated that 350,000 women died as a result of this disease [20]. In 36 countries, cervical cancer is the primary cause of cancer death. Most of these countries are located in the regions of Sub-Saharan Africa (24%), South America (16%), and Southeast Asia (14%) [20]. Figure 1A shows a diagram of the global health problem of cervical cancer associated with HPV infection based on the statistics mentioned above.

When it comes to detecting HPV and cervical cancer, two distinct approaches can be taken to address the issue. The first is through the direct detection of the HPV genome, which is usually done by amplification of nucleic acids tests (NAATs) such as Polymerase Chain Reaction (PCR). The second way involves identifying morphological changes caused by HPV infection in the cells or tissue of the patient, providing insight into precancerous or cancerous conditions. The most commonly used techniques in this field are cytology, immunohistochemistry, and Colposcopy [4,6].

The cytology technique indicates the presence of abnormal cervical epithelial cells [4,6]. Although cytology is the conventional detection method used worldwide, it has significant limitations, such as low sensitivity and trained personnel to collect the sample and expert pathologists, which is why errors in interpretation can occur and false negative results can be generated [4,6]. In comparison, immunohistochemistry tests are based on the presence of cancer biomarkers (proteins) but cannot provide a preventive diagnosis. In the case of colposcopy is a procedure to examine the cervix, the vaginal wall, and the vulva but like cytological and immunohistochemical tests; it does not provide preventive results since it identifies pre- or cancerous tissue phenotypes. Furthermore, the test is prone to errors of interpretation [20]. Finally, qPCR can be used to detect HPV RNA or DNA, having a high sensitivity and specificity, in addition to allowing the identification of the viral genotype present in the sample [21,22]. However, the qPCR requires a thermocycler (expensive equipment) and specialized laboratories to process the sample and run the test to amplify the DNA region of interest using different temperature cycles (Figure 1B shows the two approaches used for directly detecting HPV and the effects on cells or tissues caused by HPV infection, in addition to the most common methodologies used).

Thus, alternatives, called isothermal nucleic acid amplification techniques (INAATs), have emerged. These alternatives are fast and do not require sophisticated laboratories or highly qualified personnel. INAATs allow the DNA region of interest to be amplified at a constant temperature (without the need to use a thermocycler) in less than 60 min, visualizing the results using electrophoresis gel, colorimetric, turbidimetry, or fluorescence signals [21,22]. There are also variants of these technologies since they are usually accompanied by microfluidic techniques, clustered regularly interspaced palindromic repeats (CRISPR-Cas), lateral flow assay (LFA), and biosensors to provide more accurate results with a lower limit of detection (LOD) [21,22].

This review presents a comprehensive overview of the current INAATs developed for HPV detection, detailing each technology’s description, target genes, LOD, and detection methods. Additionally, we highlight the HPV tests currently available on the market and FDA-approved. The challenges that this type of test still faces in terms of large-scale use at the international level are also discussed.

## 2. Molecular Testing Applied to the Global Strategy 90-70-90

In 2020, the World Health Organization (WHO) launched a global strategy called “90-70-90” aimed at eliminating cervical cancer by 2030 [23]. The strategy is based on three pillars: the first involves achieving vaccination of 90% of girls by age 15 years, the second pillar focuses on the subject addressed in this review, which pertains to implementing high-performance testing to cover 70% of women, and the third pillar aims to ensure that 90% of women who positive test or cervical injury can be treated appropriately [23]. The implementation of the 90-70-90 strategy is projected to avert 60 million cases of cervical cancer and prevent 45 million deaths in the next 100 years. Moreover, it encompasses a broader spectrum of other preventable cancers and diseases through HPV vaccination and high-performance testing [23]. It is important to note that pillars two and three of the strategy to eliminate cervical cancer have diagnosis as their central axis. Therefore, these pillars depend on having a diagnostic tool that is accurate enough to achieve the objective. Also, seven criteria stipulated by the WHO in 2003 must be taken into account to guarantee effective tests for the diagnosis of infectious diseases such as HPV, which are affordable, sensitive, specific, user-friendly, rapid and robust, equipment-free, and deliverable to end users, which together correspond to the acronym ASSURED [24].

In addition, the COVID-19 pandemic highlighted the need for highly accurate tests that are quickly implementable in the field and low-cost to control the spread of pathogens [25]. The qPCR was widely used to detect SARS-CoV-2, and this sensitive technique was standardized in numerous countries within specialized laboratories [26]. Unfortunately, not all countries or regions have these specialized laboratories, and the main limitation of the qPCR technique is the thermocycler, an expensive equipment used for fluorescence detection and to provide different temperatures required for the polymerase to amplify nucleic acids [26].

**Figure 2:**
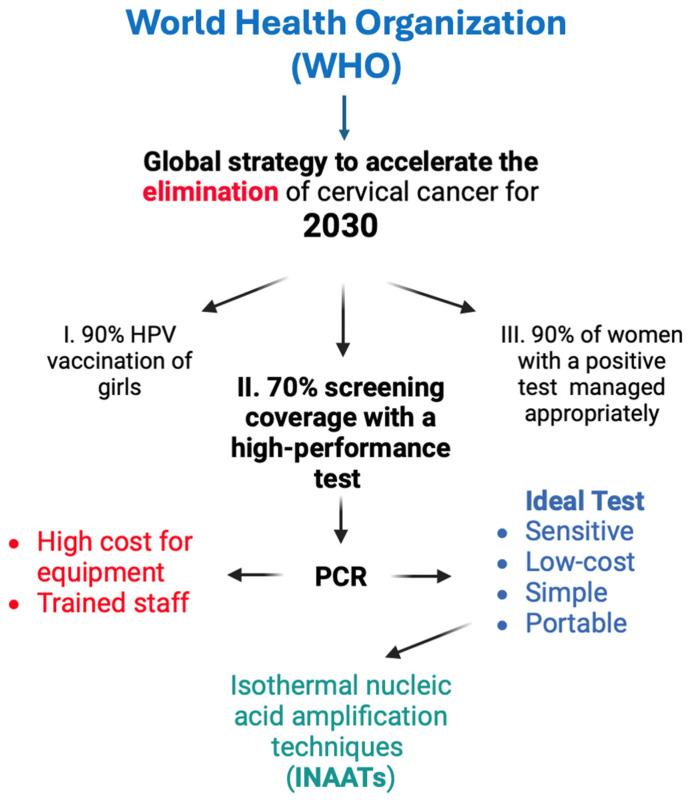
The global strategy 90-70-90 established by the World Health Organization (WHO) is composed of three essential pillars. Diagnosis is a core point for the strategy, where Isothermal nucleic acid amplification techniques (INAATs) can be a powerful tool in the fight against cervical cancer and HPV [18].

To achieve tests that can be used on a large scale in remote or precarious areas, it is necessary to work mainly in two ways: (a) reduce the price of thermal cyclers to massify qPCR tests, or (b) develop and implement new nucleic acid amplification methods, such as isothermal tests methods that can be conducted using simple and low-cost equipment. These mentioned techniques offer the advantage of being isothermal and avoiding using a thermocycler. Figure 2 illustrates explicitly the strategy 90-70-90 and its correlation with timely and sensitive diagnosis to attain the objective.

## 3. Isothermal Tests for HPV Detection

INAATs have emerged as an alternative to qPCR for detecting several pathogens, including HPV [27]. These tests have multiple advantages over qPCR since they are considered fast (approximately less than 60 min), allowing the DNA amplification reaction to be carried out at a constant temperature (without the need for thermocyclers) to detect the presence of HPV [21,22]. It is also important to highlight that they are easy to use, allowing samples to be analyzed on a large scale, and most of them do not require prior sample processing [21,22].

**Figure 3:**
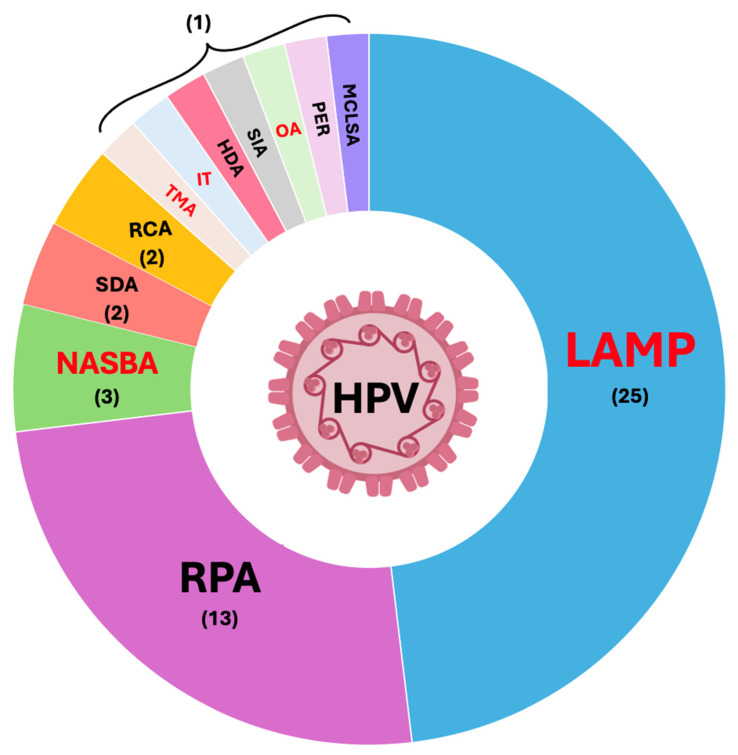
A graph of the INAATs developed and published in the literature used to detect HPV. The number of tests developed is indicated, and the techniques marked in red indicate a commercial product based on it for the detection of HPV.

Among the main INAATs reported in the literature that are used for HPV detection are loop-mediated isothermal amplification (LAMP), recombinase polymerase amplification (RPA), rolling circle amplification (RCA), transcription-mediated amplification (TMA), invader technology (IT), helicase-dependent amplification (HDA), self-primed isothermal amplification (SIA), omega amplification (OA), primer exchange amplification (PER), cross-linking spiral amplification (MCLSA), nucleic acid sequence-based amplification (NASBA), and strand displacement amplification (SDA) (Figure 3).

These technologies usually use detection methods such as electrophoresis gels, which allow the band of interest to be observed; turbidity derived from magnesium pyrophosphate precipitation due to the amplification reaction, visualized with the naked eye [27,28,29]. pH-sensitive colorimetric methods are also used, such as phenol red and cresol, which, through a change from pink to yellow with the naked eye, indicate the presence of the target sequence; this is a consequence of the decrease in pH due to the release of protons (hydrogen ions), after the amplification reaction [30,31]. Another dye that, with the naked eye, allows visualizing the presence of HPV is hydroxynaphthol blue (HNB); when it changes from violet to sky blue after the loss of the magnesium ions attached to HNB to interact with the pyrophosphate derived from the amplification reaction of the target sequence, producing magnesium pyrophosphate an insoluble form [32,33,34]. Also, the eriochrome black T (EBT) dye uses the same basis as HNB, changing color (red to blue) after the loss of magnesium ions, the hue of EBT being three times more intense than HNB [35]. Likewise, nanoparticles have been used that are sensitive to salts that change color from blue (absence of virus) to red (presence of the virus) [36].

Fluorescence signals have also been utilized using fluorophores such as calcein, SYBR green I, and EvaGreen. When excited with UV or blue light, fluorescence indicates the target virus’s presence [37,38,39,40,41,42]. Calcein, in the absence of the target sequence, is bound to magnesium ions, which act as a quencher, but in the presence of the virus, the region of interest is synthesized, and magnesium binds to pyrophosphate, releasing calcein and emitting a fluorescence signal when excited with UV light [37,38,39]. Regarding SYBR Green I and EvaGreen, after amplifying the target sequence, these are intercalated within the double-stranded DNA, emitting a green fluorescent color when excited with UV or blue light [40,41,42].

**Figure 4:**
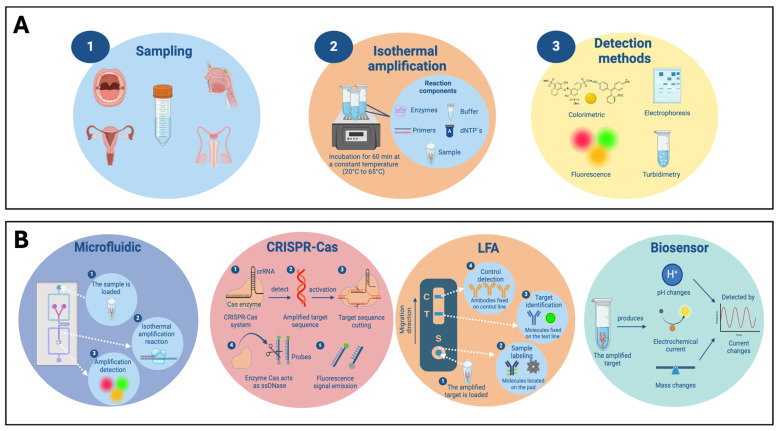
Representative scheme of the general methodology to carry out INAATs and the platforms with which they are usually combined. (**A**) The steps to be followed for amplification of the target region of the HPV virus using INAATs are shown. (1) The first step consists of taking a sample of the tissue of interest (mouth, cervical, oropharynx, penis, anus, urine, among others); (2) The sample is subsequently incubated with the components of the amplification reaction (enzymes, buffer primers, and dNTPs) at a constant temperature (ranging from 20 °C to 65 °C) for approx. 60 min. (3) The presence of the virus can be detected by electrophoresis, turbidimetry, fluorescence, or colorimetric methods. (**B**) Furthermore, INAATs are combined with different platforms such as microfluidic techniques that allow the sample to be loaded into the device (1), to later carry out the amplification reaction on a chip (2) and detect the presence of HPV (using colorimetric or fluorescence methods) (3). Also, the CRISPR-Cas system has been used (1), which detects the target sequence by crRNA (2), causing a cut in the target sequence (3), and activating the Cas enzyme which acts as a ssDNase cutting the probes conjugated with fluorophores and quencher (4), and releasing a fluorescence signal indicating the presence of HPV (5). Likewise, lateral flow assay (LFA) has been used, in which after the amplification reaction, the sample is placed on the strip (1), and is labeled with molecules or antibodies located on the sample pad (2), and through the appearance of one band on the test line and another on the control line, it detects HPV since the labeled sample interacts with the molecules or antibodies fixed in the test (3) and control (4) lines. Also, biosensors that identify HPV’s existence through mass, voltage, or electrochemical current changes.

In addition, INAATs are usually accompanied by microfluidic, CRISPR-Cas, lateral flow assay (LFA), and sensor technologies (Figure 4).

Microfluidic devices are considered lab-on-a-chip, since through well-controlled environments, which use the manipulation of fluids in channels of sizes from nano to micrometers, allowing the handling and analysis of the sample of interest by separating it and incubating it with the components of the amplification reaction, and producing a signal that indicates the presence of the target of interest [43]. These types of technologies increase precision and sensitivity and reduce human errors [43]. Besides, the CRISPR-Cas system consists of an RNA (crRNA) sequence designed to detect the target region; a Cas nuclease (such as Cas9, Cas13a, Cas12a, Cas12b, and Cas14); and a single-stranded DNA (ssDNA) or aptamer reporter sequence that is labeled with a fluorophore and quencher [22]. In the presence of HPV, the RNA sequence guides the CRISPR-Cas complex to the target, and the Cas enzymes cut the region of interest. In addition, the Cas enzymes act as a non-specific ssDNase, making non-specific cuts in the reporter ssDNA or aptamer sequence, releasing a fluorescence signal [22].

Regarding LFA, this technology is usually used to reveal the INNATs’ reactions and allow for a quick interpretation of the results through colorimetric changes. This type of assay is based on the use of labeled probes (FITC and FAM) or primers conjugated with fluorophores (FITC and FAM) and biotin [44,45,46]. On the other hand, INAATs coupled to biosensors allow the presence of the HPV target genotype to be indicated by detecting pH, electrochemical, or mass changes [47,48,49].

### 3.1. LAMP

LAMP is one of the isothermal platforms that has been most exploited for detecting HPV DNA and other pathogens. This technique was invented in 2000 by Notomi and collaborators [50]. LAMP is based on the use of an isothermal polymerase *Bst* (with high strand displacement activity) from *Geobacillus stearothermophilus*, which works at a temperature between 60–65 °C (employing thermoblock and water baths) and uses at least four primers that recognize six different regions of the target sequence [22,50]. One of the pairs of primers is called inner primer, which consists of forward inner primer (FIP) and backward inner primer (BIP); each primer contains two different sequences, an antisense sequence and another sense of the target sequence [50]. This last sequence will allow the loop to form when the target DNA strand self-hybridizes after the amplification reaction [50]. The second pair of primers are called outers and are composed of forward outer primer (F3) and backward outer primer (B3) [50]. The LAMP reaction occurs in less than one hour and is compatible with DNA or RNA sequences.

The LAMP technique uses different detection methods, which can be conventional (electrophoresis, turbidity, colorimetric, or fluorescence) or can be accompanied by other platforms such as microfluidic, LFA, or biosensors (Table 1).

#### 3.1.1. Conventional Detection Methods

The detection of the HPV target sequence usually uses electrophoresis gels and turbidity [28,29]; a clear example was developed by Yang et al. (2016), who used the LAMP technique to amplify HPV (5, 8, 16, 18, and 58 genotypes) and herpes simplex virus-2 (HSV-2), at a temperature of 63 °C in a time of 45 min. Using agarose gels and turbidity, they determined the presence of the target viruses. This technique presents a LOD of 10 copies/μL and 10^2^ copies/μL for HPV and HSV-2, respectively [29].

Also, pH-sensitive dyes have been used for the detection of high-risk HPV genotypes (16 and 18) from cervical samples and cells in oral rinses, with primers that amplify the gene E6 at 65 °C in a time of 15 to 80 min [30,31]

Another dye used is HNB, and an example was reported by Zhu et al. (2022), who employed the nano-LAMP technique to identify the HPV-6 genotype of genital wart samples at a temperature between 64–65 °C, which was obtained through an internal heat source, which consists of magnetic nanoparticles (FE_3_O_4_), excited at a wavelength of 808 nm near-infrared light. In the presence of HPV-6, the color change from violet to sky blue was observed after an incubation of 60 min, and the test presented a LOD of 10^2^ copies/mL [34].

Similarly, EBT dye has been used to identify the presence of HPV-16 after amplification of the target sequence at 63 °C in a time of 60 min in saliva and cervical swab samples, and this technique has a LOD of 10^4^ copies per reaction [35].

In addition, gold nanoparticles (AuNP) anchored to probes that recognize the target sequence have been used. When magnesium salt is added, a color change from blue to red occurs in the presence of the target HPV genotypes; the salts turn the charge of the AuNPs surface in neutral, stabilizing the conjugates (AuNP-target sequence), causing their aggregation [36]. In the absence of the target virus, the blue color is maintained due to the instability of AuNP with the probe. Kumvongpin et al. (2016), developed this technique to identify HPV-16 and HPV-18 genotypes from cervical tissue samples, which, in the presence of these genotypes, produced a color change from blue to red after incubation at 65 °C for 20 min, with a LOD spanning a range of 1 to 10^5^ copies/reaction [36].

Fluorescence signals have also been used to detect the HPV target sequence using fluorophores such as calcein, SYBR green I, and EvaGreen [37,38,39,40,41,42]. Different research groups have used calcein in LAMP to detect the presence of HPV genotypes that cause cancer (16, 18, 23, 33, 39, 45, 52, and 58); from cervical samples, amplifying regions of the E7 and L1 genes, at 65 °C for one hour [37,38,39]. On the other hand, the SYBR Green I fluorophore in LAMP has been used to detect genotypes 16 and 18 from saliva samples, tumor tissues in the mouth, blood, and cervical tissues by amplifying the regions of the E7 and L1 genes at a temperature of 60 °C for 60 min [40,41].

Furthermore, to carry out studies with greater precision that allow the early detection of cervical cancer, Wormald et al. used LAMP in combination with endovaginal magnetic resonance imaging (MRI). In this context, the LAMP amplification reaction was initially conducted at 64 °C for 45 min to detect the E7 and L1 genes/transcripts of HPV-16 and HPV-18 and the presence of tumor biomarkers hTERT, TERC, and c-MYC mRNAs (performing reverse transcription of RNA molecules and amplification); from cervical cytological swab samples. This assay had a LOD of 10^2^ to 10^4^ copies/reaction. The LAMP results were combined with MRI image results to provide an early diagnosis of cervical cancer and make decisions about the appropriate surgical procedure for patients who wish to preserve fertility [51].

#### 3.1.2. Microfluidic-LAMP

Also, microfluidic platforms have been combined with LAMP to detect different HPV genotypes (Table 1). A clear example of the use of microfluidic technology was reported by Bai et al. (2024), who developed a microfluidic-LAMP device to identify the presence of HPV-16 and HPV-18 from cervical swabs [42]. This device considered a portable all-in-one device (PAD), allowed the sample to be lysed and the LAMP reaction to be carried out using lyophilized reagent beads and, in the presence of the virus, the E6, E7, and L1 genes were amplified, releasing a fluorescent signal (by EvaGreen) after 15 min of incubation at 65 °C, with a LOD of 1 copy/μL [42].

Microfluidic-LAMP platforms have also been developed for detecting 14 high-risk HPV genotypes through a self-digitization chip, which automatically digitizes the sample in a well array (volume in nanoliters) of DNA purified from urine samples and of cervical and vaginal swabs, which in the presence of any of the target genotypes (16, 18, 31, 33, 35, 39, 45, 51, 52, 56, 58, 59, 66, and 68) emit a fluorescence signal (by calcein), after the synthesis of some region of the HPV genome or the L1 gene, in a time of 47 min at 65 °C. This technology presents an LOD of 10 to 10^3^ copies/reaction [52]. Likewise, for the interpretation of the results of the microfluidic-LAMP platforms, smartphones that use applications such as Hue Analyzer have been used to detect genotypes 16, 18, and 31 from saliva and cervical swab samples through colorimetric changes of HNB [53]. In addition, microfluidics-LAMP systems have been used, which, through droplets, carry out the LAMP reaction to detect and quantify HPV-16 and HPV-18 from cervical swab samples by amplifying regions of the E7 and L1 genes in a time of 60 min at 63 °C, releasing a fluorescence signal (by calcein) in the presence of any of these genotypes [54].

#### 3.1.3. LFA-LAMP

Regarding LFA-LAMP, the LAMP reaction is first carried out, and in the presence of HPV, products labeled with biotin are obtained and subsequently hybridized with a probe that contains a fluorophore or another alternative to generate doubly labeled amplicons (primers conjugated with fluorophore and biotin) (Table 1) [44,45,46]. Once the LAMP reaction is completed, the sample is loaded on the sample pad of the strip and conjugated with anti-FAM antibodies or molecules (neutravidin or streptavidin) labeled with nanoparticles (gold or carbon) [44,45,46]. Subsequently, the sample migrates, and in the test line, anti-fluorophore antibodies (FAM or FITC), or avidin, are fixed to bind with their corresponding conjugates, generating a red or purple-red band (due to the nanoparticles). The rest of the nanoparticles that are not conjugated migrate to the control line where anti-rabbit or anti-streptavidin antibodies are fixed and interact with anti-fluorophore antibodies or avidin, observing a red or purple-red line. In the absence of the virus, only the control line is observed [44,45,46]. Among some examples of this technology is the LFA-LAMP developed by Landaverde et al. (2020), who amplified and labeled the amplicons derived from the E7 gene with 6-FAM and biotin to identify the presence of HPV 16 genotype from DNA samples of cervical swabs, incubating at 63 °C for 60 min [44]. The revelation of the LAMP product was carried out on a commercial strip that contains streptavidin-tagged gold nanoparticles in the sample pad and anti-FAM antibodies in the test line, and in the control line, there are anti-streptavidin antibodies, yielding results in 10 min [44].

LFA-LAMP is also available to detect two HPV genotypes (16 and 18) using a FITC-conjugated probe that hybridizes with biotin-labeled amplicons (biotin-conjugated inner primers) produced in a LAMP reaction at 65 °C for 30 min. Using a commercial strip, the LAMP product is revealed, which in the sample pad contains anti-FITC antibodies labeled with gold nanoparticles, and in the test line avidin molecules are fixed, and in the control line, anti-rabbit antibodies are fixed. This test has a LOD of 1 to 10^5^ copies/reaction, and the results are observed in 5 min [45].

Likewise, a total of 24 primers were used in a reaction to determine the presence of HPV-16, HPV-18, and HPV-45 by labeling the amplicons with FAM and biotin derived from the E6, L1, and E7 genes, which are obtained after incubation at 65 °C for 30 min [46]. In addition, positive amplification controls have been used with B-actin labeled with digoxigenin (DIG) and biotin [46]. The amplicons are loaded on the commercial strip, which in the sample pad contains carbon nanoparticles coated with neutravidin and two test lines: in the first one are anti-DIG antibodies, the second test line is anti-FAM antibodies, and the control line is anti-biotin antibodies. This test had a LOD of 10 to 10^2^ copies/reaction, and the results were obtained in 10 min [46].

#### 3.1.4. LAMP Integrated with Biosensors

Furthermore, LAMP combined with biosensors allows the presence of the HPV target genotype to be indicated by detecting pH, electrochemical, or mass changes (Table 1). As for the sensors that identify pH changes, Wormald et al. developed a device that consists of a Complementary Metal-Oxide-Semiconductor (CMOS) technology in conjunction with Ion-Sensitive Field-Effect Transistor (ISFET) sensors [47,48,49]. This device allows the LAMP reaction to be carried out by amplifying the DNA of HPV-16 and HPV-18. Further, the mRNA of the hTERT (tumor biomarker) from cervical cancer tissue samples and through voltage changes generated by the release of hydrogen ions (after the amplification reaction), the presence of HPV is determined. This sensor has a LOD of 10 to 10^2^ copies/reaction, and the results can be observed in less than 25 min, sending them to the smartphone via Bluetooth [47].

Regarding electrochemical biosensors, Yang et al. generated a sensor that can determine the presence of HPV-16 from the reverse transcription and amplification of E6 and E7 mRNA, labeled with biotin-11-duTP, from cervical cells. Once the target regions have been amplified (at 63 °C for 45 min), these are incubated with gold nanoparticles that have anchored probes that hybridize with the amplified product. These conjugates are used to manufacture the graphite electrode, to later bind with streptavidin labeled with horseradish peroxidase, and in the presence of 3,3′,5,5′-tetramethylbenzidine (TMB), generate an electrochemical current. This biosensor has an LOD of 10^2^ copies [48].

On the other hand, to detect HPV genotypes through mass changes, Prakrankamanant et al. developed a quartz crystal microbalance that detects the presence of HPV-58 from cervical samples. In this sense, avidin is fixed to the surface of the microbalance, which interacts with the LAMP products (63 °C amplification reaction), biotinylated (by primers inners forward labeled with biotin). After the interaction, a change in frequency is generated on the surface of the microbalance due to mass variations. This device indicates results in less than 30 min and has a LOD of 10^2^ copies [49].

**Table 1:** LAMP tests were developed for the detection of different HPV genotypes.

LAMP Test Type	Sample (Tissue)	Genotype	Target Gene	LOD	Detection Signal	Detection Time (min)	References
Conventional	Genital polypoid	6-11-16-18	E6-E7	10^3^ copies/reaction	Turbidity	59	[55]
	Oropharyngeal	16-18-31-33-35	-	-	Turbidity	60	[27]
	Cervical	5-8-16-18-58	L1-L2	10 copies/μL	Turbidity	45	[29]
	Cervical	16-18-45-58	-	10^2^ to 10^3^ copies/reaction	Turbidity	60	[28]
	Cervical	16-18	E6	10 copies/reaction	Colorimetric (Phenol red)	<80	[30]
	Oral rinses	16-18	E6	10 cells	Colorimetric (Phenol red)	20	[31]
	Cervical	16-18-45-52-58	E6-E7-L1	10 to 10^2^ copies/reaction	Colorimetric (HNB)	65	[32]
	Cervical	6-11-16-42-43-44	E6-E7-L1	10^3^ copies/reaction	Colorimetric (HNB)	65	[33]
	Cervical	6	-	10^2^ copies/mL	Colorimetric (HNB)	60	[34]
	Cervical	16-18	E7-L1	10 copies/reaction	Fluorescence (SYBR Green-I)	60	[40]
	Oral Saliva Blood	16	E7	46.8 copies/μL	Turbidity and Fluorescence (SYBR Green-I)	23	[41]
	Cervical	16-18	E7-L1	10^2^ cells/mL	Fluorescence (Calcein)	70	[37]
	Cervical	16-18-33-39-45-52-58	-	20 copies/μL to 20^5^ copies/μL	Fluorescence (Calcein)	19–75	[39]
	Cervical	16-18	-	10 copies	Colorimetric (Gold nanoparticles)	25	[36]
	Cervical	16-18	E7-L1	1 to 10^4^ copies/reaction	---	45	[51]
Microfluidic	Cervical	16-18	E6-E7-L1	1 copy/μL	Fluorescence (EvaGreen)	15	[42]
	Urine Cervical Vaginal	16-18-31-33-35-39-45-51-52-56-58-59-66-68	L1	10 to 10^3^ copies/reaction	Fluorescence (Calcein)	47	[52]
	HUVEC and HeLa cells	16-18-39-45-52	-	10^3^ to 10^6^ copies/μL	Fluorescence (EvaGreen)	40	[56]
	Plasmid DNA	18	L1	150 copies/μL	Fluorescence (Calcein)	80	[57]
	Skin warts	1-2-3-4-5-7-8-9-10-12-14-27-28-29-41-48-49-50-57-63-65-75-76-77-94-95-115-117-125-160	L1	10^7^ copies/μL	Colorimetric (HNB)	60	[58]
	Saliva Cervical	16-18-31	-	50 copies/reaction	Colorimetric (HNB)	60	[53]
	Cervical	16-18	E7-L1	-	Fluorescence (Calcein)	60	[54]
	Saliva Vaginal	16	-	10^2^ copies/reaction	Colorimetric (EBT)	60	[35]
LFA	Cervical	16	E7	-	Colorimetric (Gold nanoparticles)	60	[44]
	Cervical	16-18	-	1 to 10^5^ copies/reaction	Colorimetric (Gold nanoparticles)	35	[45]
	Cervical	16-18-45	E6-L1-E7	10 to 10^2^ copies/reaction	Colorimetric (Neutravidin-coated carbon nanoparticles)	40	[46]
Biosensor	Cervical	16-18	E6-E7-L1	10 to 10^3^ copies/reaction	Voltage	25	[47]
	Cervical	58	-	10^2^ copies/mL	Mass changes	30	[49]
	Cervical	16	E6-E7	10^2^ copies/mL	Electrochemical	<120	[48]
	Cervical	16-18	E6-E7	1 ng/reaction	Electrochemical	90	[59]
	Cervical	16-18	E6-E7	10 cells/reaction	Electrochemical	45	[60]

LOD (Limit of detection), EBT (eriochrome black T), HNB (hydroxynaphthol blue).

### 3.2. RPA

Recombinase polymerase amplification (RPA) was developed in 2006 by Olaf Piepenburg et al. This isothermal technique consists of the use of a pair of primers, a recombinase protein, single-strand binding (SSB) proteins, and DNA polymerase. The amplification reaction is carried out at a temperature of 20–42 °C in an approximate time of 60 min [61]. The RPA reaction begins when the recombinase forms a complex with the primers, searching for homologous sequences, allowing the primers to bind to their complementary sequences, forming a D-loop structure, and the SSB proteins stabilize the single strand displaced by the recombinase to avoid the removal of the primers. Finally, the DNA polymerase removes the recombinase, carrying out the synthesis of the strands complementary to the template [62,63].

For the detection of HPV genotypes, different types of tests have been developed that use RPA, which observes the presence of the virus through electrophoresis, fluorescence signals, or, when RPA is accompanied by techniques such as microfluidics, it observes LFA, dot blot, or biosensors (Table 2).

#### 3.2.1. Conventional Detection Methods

Among the RPA methods that use electrophoresis is the one reported by Wongsamart et al. (2023). These researchers performed multiplex RPA to identify 20 high-risk genotypes (16, 18, 26, 31, 33, 34, 35, 39, 45, 51, 52, 53, 56, 58, 59, 66, 68, 69, 73, and 82) and 14 low-risk HPV genotypes (6, 11, 32, 40, 42, 43, 44, 54, 61, 70, 72, 81, 84 and 87). The study used degenerated primers that hybridized in regions of the E6, E7, and L1 genes from cervical swab samples. The amplification reaction was carried out at 39 °C for 40 min, and to stop the reaction, the sample was incubated for 2 min at 80 °C. The results were visualized by electrophoresis, with the test presenting an LOD between 10^2^ and 10^3^ copies [64].

For this study, regarding RPA assays using fluorescence signals, ROX- and FAM-labeled probes were developed for single and dual detection of HPV-16 and HPV-18, which are specific for the E7 (single assay) and L1 (dual assay) genes from DNA obtained from cervical samples. The RPA amplification reaction was carried out at 37 °C for 25 min. This test has a LOD of 50 to 10^3^ copies/μL (single assays) and 10^4^ copies/reaction for dual assays [65].

#### 3.2.2. RPA-CRISPR-Cas and Microfluidic

The RPA technique has also been accompanied by CRISPR-Cas to detect HPV by fluorescence. These techniques have been used mainly to determine multiple high-risk HPV genotypes (6 to 13), employing universal or degenerate primers and performing the amplification reaction at 37 °C for 20 min. After the RPA reaction, the sample is incubated with the CRISPR-Cas system at 37 °C for 5 to 20 min, and in the presence of HPV, a fluorescence signal is emitted. These techniques have an LOD of 1 copy/μL to 40 copies/μL (Table 2) [66,67].

Also, RPA, CRISPR-Cas, and microfluidics have been combined to detect HPV (Table 2). Among them is the platform called MiCar (CRISPR-Cas12a and multiplex RPA) proposed by Xu et al. (2022), which allows the detection of 9 HPV genotypes (6, 11, 16, 18, 31, 45, 52, and 58) using specific primers and crRNA for each subtype that recognize regions of the L1 gene, using the same fluorophore for all genotypes. This microfluidic device consists of a central entrance where the sample amplified by RPA is loaded. The central input is connected to 30 outputs, which are wells that are preloaded with different crRNAs (each cRNA is in triplicate), a reporter, and the enzyme Cas12a, which recognizes one of the 9 target genotypes. This test has an LOD of 0.26 aM and takes 40 min [68].

Unlike MiCar, Yin et al. generated a microfluidic platform that consists of 3 chambers that are inserted in a well of a 96 plate to carry out both the RPA reaction and the detection by CRISPR-Cas12a in the same system without the need to separate operations, employing a dynamic aqueous multiphase reaction (DAMR) system, which is based on a difference in sucrose concentration density, spatially separating RPA (located in the high-density phase, at the bottom of the well) and the CRISPR-Cas12a system (located in the low-density phase, at the top of the well), which, in turn, are connected by an aqueous phase. This system contains a control chamber (without crRNA) and two chambers with a specific crRNA to identify HPV-16 or HPV-18 from cervical swab samples. This platform has a LOD of 10 to 10^2^ copies and provides results in less than one hour after incubation at 37 °C [69].

Another attractive microfluidic platform that uses the CRIPR-Cas13a system for detecting HPV was developed by Cui et al. (2022). This platform called PADLOCK (whose acronym stands for pico-injected, aided digital reaction unlocking) generates digital droplet RPA (ddRPA) to quantify viral load. The device consists of a pico-injector and a droplet generator. First, emulsified droplets deprived of MgOAc (to avoid premature amplification) containing master mix (components for the RPA reaction and CRISPR-Cas13a system) are generated to perform the amplification of the L1 region of HPV-16 from cervical samples; subsequently, these drops are injected with MgOAc, using a pico-injector. Finally, the sample is incubated at 37 °C for 30 min, and in the presence of HPV-16, a fluorescence signal is emitted; this platform has a LOD of a single viral molecule [70].

Fluorescent microfluidic platforms that do not use the CRISPR-Cas system have also been developed. An example was reported by Khamcharoen et al. (2023) to detect HPV-16. This capillary-driven microfluidic circuit is based on stacked layers of transparent films and double-sided adhesive films, which allow the flow to be driven by generating a capillary in the gap between the space of the two layers of the film and through the difference in height of the channel, a valve is created that stops the flow of the fluid, allowing the RPA reaction to be carried out at 38 °C in 30 min. The valve is then pressed, and the amplified product is transferred to the detection system and mixed with SYBR Green I, generating a fluorescent signal after amplifying the L1 target region of HPV-16. This circuit has a LOD of 0.24 pg/μL [71].

#### 3.2.3. LFA-RPA

Systems based on LFA that incorporate RPA have been used to detect low- and high-risk HPV genotypes. These assays consist of first carrying out the RPA amplification reaction with primers labeled with biotin, hapten, digoxin, or FITC, which recognize target regions of the E6, E7, or L1 genes after incubation between 37 to 39 °C for a time of 10 to 30 min. The RPA products are placed on the sample pad of the strip and conjugated with antibodies labeled with nanoparticles or streptavidin-coated nanostructures. Through capillary action, the samples reach the test line where capture oligos, streptavidin, or anti-FITC or anti-Digoxigenin antibodies are fixed to identify the presence of the amplified products. An anti-antibody antibody (with or without biotin) or biotinylated bovine serum albumin is fixed in the control line. These types of tests take 30 to 60 min. In the presence of the target genotypes, two (identifies one genotype and the control) to three (more than one genotype and the control) lines can be seen on the strip. In the absence of the genotypes of interest, only one line is observed that corresponds to the control (Table 2).

To improve the specificity of the RPA reaction before visualizing the results on LFA, graphene oxide has been used to enhance the match of the primers with their template and avoid mismatch that generates non-specific products, which allows eliminating experimental tests of optimization of primers and use of probes. For this assay, a concentration of 56 μg/mL of graphene oxide was used to carry out the RPA reaction with primers labeled with FITC (HPV-16) and digoxigenin (HPV-18) that recognize regions of L1 for the detection of HPV at a temperature of 38 °C for 30 min. After the reaction, the sample was loaded into an LFA system, conjugating with nanoparticles coated with the crimson red dye and streptavidin. When HPV is present, the anti-FITC antibodies in the first test line interact with the amplified region of HPV-16, while in the second test line, there are anti-digoxigenin antibodies that recognize HPV-18. This LFA has a LOD of 10 copies/reaction, and the results were observed in 60 min [72].

RPA has also been accompanied by techniques such as reverse dot blot, which is based on the use of probes fixed by covalent bonds on a nylon membrane; a clear example was developed by Ma et al. (2019), who first performed an RPA reaction to amplify conserved regions of the L1 gene of low- and high-risk HPV genotypes, using biotin-labeled primers. The RPA reaction was carried out at 37 °C for 10 min; in the presence of the biotinylated target, it hybridizes with the fixed probes specific for L1, and subsequently, streptavidin conjugated with horseradish peroxidase is added, which in the presence of TMB, a purple-blue color is observed [73]. This test has an LOD of 10^3^ copies/μL and takes about 45 min. Likewise, Ma et al. performed an LFA test after the RPA reaction with primers labeled with biotin and digoxin; the amplified sample in the LFA was conjugated with anti-digoxin antibodies labeled with colloidal gold, and the biotinylated products were recognized by streptavidin fixed on the test line and the control line had anti-body antibodies. This LFA system has an LOD of 100 fg, and the results are observed after 5 min [73].

#### 3.2.4. RPA Integrated with Biosensors

Also, RPA has been combined with biosensors to identify HPV-16. These can be made up of probes immobilized to the electrode that, in the presence of the amplified ones that correspond to regions of the E6/E7 genes, hybridize with the probes, incubating these products with methylene blue, which is intercalated in the double strand. Generating an oxidation reaction on the electrode, the signal is measured by differential pulse voltammetry. This device has a LOD of 0.23 copies/μL, and the results take 75 min (Table 2) [61].

On the other hand, Li et al. (2021) used a biosensor free of immobilization, and to achieve this, they incorporated the CRISPR-Cas12a technology after RPA amplification of HPV-16, using specific primers for the L1 gene, the CRISPR-Cas12a system was activated, which led to cleavage of the reporter ssDNA labeled with methylene blue. The reporter was detected by the negatively charged electrode surface since when the cut is made on the ssDNA, smaller fragments are generated, and the negative charge decreases, which allows the fragmented ssDNA to spread freely over the surface of the electrode, generating an increase in the electrochemical current. Meanwhile, in the absence of the target, the negative charge of the ssDNA-MB is greater, and it is repelled by the electrode surface, producing a low electrochemical current. This system has a LOD of 1 pM, and results are observed in less than 120 min (Table 2) [74].

**Table 2:** RPA tests were developed and published for the detection of different HPV genotypes, with the main characteristics.

RPA Test Type	Sample	Genotype	Target Gene	LOD	Detection Signal	Detection Time (min)	References
Conventional	Cervical	6-11-16-18-26-31-32-33-34-35-39-40-42-43-44-45-51-52-53-54-56-58-59-61-66-68-69-70-72-73 -81-82-84-87	E6-E7	10^2^ to 10^3^ copies/reaction	Electrophoresis	40	[64]
	Cervical	16-18	E7-L1	50 to 10^3^ copies/μL	Fluorescence (FAM and ROX)	25	[65]
CRISPR-Cas	Cervical	16-18-31-33-35-39-45-51-52-56-58-59-68	L1	500 copies/reaction	Fluorescence	35	[66]
	Cervical	16-18-31-33-35-45	L1-E6-E7	1 to 10 copies/μL	Fluorescence (FAM)	40	[67]
	Cervical	16	L1	1 pM/reaction	Electrochemical (Methylene Blue)	<120	[74]
Microfluidic	Cervical	6-11-16-18-31-45-52-58	L1	0.26 aM/reaction	Fluorescence (FAM)	40	[68]
	Cervical	16-18	-	10 to 10^2^ copies/reaction	Fluorescence	60	[69]
	Cervical	16	L1	10 to 10^2^ copies/μL	Fluorescence (FAM)	30	[70]
	Cervical	16	L1	0.24 pg/μL	Fluorescence (SYBR Green I)	30	[71]
LFA	Cell lines (C33A, SiHa, HeLa, and MS751)	16-18-45	E7	5000 to 50,000 cells/mL	Colorimetric (Gold nanoshells)	35	[75]
	Cervical	16-18	L1	10 copies/reaction	Colorimetric (Crimson red)	60	[72]
	Cervical	16-18	E6-E7	5 to 10 copies/reaction	Colorimetric (Gold nanoparticles)	30	[76]
	Cervical	6-11-16-18-26-31- 33-35-39-40-42-43-44-45-51-52-53-56-58-59-68-73-81-83	L1	0.1 to 1 pg/reaction	Colorimetric (Gold nanoparticles)	60	[73]
Biosensor	Cervical	16	E6-E7	0.23 copies/μL	Electrochemical (Methylene Blue)	75	[61]

LOD (Limit of detection).

### 3.3. NASBA and TMA

NASBA represents a pioneering isothermal technique developed in 1991 and is based on the retroviral replication process. Usually, the target molecule is RNA and initially consists of a reverse transcription that is carried out using a primer that includes the sequence of the T7 promoter and a reverse transcriptase, generating a duplex between DNA (cDNA) and RNA (target) (Table 3). After this reaction, the RNA strands are degraded using the enzyme RNase H and thus can be used to produce single strands of DNA. With a second primer, the double strand of DNA is synthesized using the same reverse transcriptase to have a DNA duplex, and the T7 polymerase synthesizes RNA exponentially, which can be coupled by the sequence added with the initial primer. This methodology involves an incubation at 41 °C for 90 to 120 min using the enzymes Avian Myeloblastosis Virus Reverse Transcriptase (AMV-RT), T7 RNA polymerase, and RNase H [77,78].

NASBA was the first isothermal methodology used for the detection of HPV RNA, being created in 1995 by Smits et al. In this work, they published a method for detecting HPV-16 RNA, in which the result is visualized by Northern blot or electrophoresis; the reported LOD is 10^2^ copies/reaction [79]. Several products utilizing NASBA technology have been introduced to the market. PreTect HPV-Proofer 7^®^ (Mel-Mont Medical, Doral, FL, USA) and NucliSENS EasyQ (bioMérieux, Craponne, France) are the most prominent and studied. Various research groups have evaluated these products under different conditions. For instance, a previous version of PreTect HPV-Proofer 7^®^, which was sold by NorChip, underwent analysis on 190 biopsies, successfully detecting HPV genotypes 16, 18, 31, 33, 45, 52, and 58. This assay relies on real-time detection of E6/E7 transcripts [79]. Likewise, there are reports of the performance of the NucliSENS EasyQ test, reporting a LOD of 5000 copies/μL of HPV. The design is also based on the real-time detection of the E6/E7 mRNA; however, they only detect genotypes 16, 18, 31, 33, and 45 [80]. These tests mainly use the detection of E6/E7 mRNA transcripts because they are considered a specific marker for cervical dysplasia and cancer. In addition, various authors have reported a specificity equal to or greater than tests based on viral DNA detection [80,81,82].

A very similar technique to NASBA is Transcription-Mediated Amplification (TMA), with the difference that it only uses two enzymes: Moloney Murine Leukemia Virus Reverse Transcriptase (M-MLV RT) and T7 RNA polymerase, avoiding the use of RNase H, since reverse transcriptase degrades the RNA template, degrading the DNA-RNA hybrids (Table 3) [78].

It is essential to note that NASBA and TMA are the exclusive INAATs utilized for detecting HPV RNA, whereas the most prevalent method involves detecting HPV DNA.

APTIMA HPV Assay is one of the most studied and referenced commercial products in the literature and is based on TMA technology. In addition, it is one of the few approved by the FDA; two variants of this test are marketed by Gen-Probe (bioMérieux, Craponne, France) [83].

Han et al. report the comparison between the APTIMA HPV Assay and a p16 oncoprotein immunohistochemistry in 50 samples analyzed; they declare a concordance of 92% in the results [84]. In another work, the comparison was made with the Hybrid Capture^®^ test, which is based on the detection of viral DNA. In this work, SiHa cells were used as a positive control, in addition to plasmid DNA (HPV-16) and RNA (E6/E7 mRNA), which reported a concordance of 94.2% between the tests. The LOD of the APTIMA^®^ HPV Assay mentioned is 10^3^ copies/mL (equivalent to 10^2^ copies/reaction) [85]. Finally, one of the most interesting comparisons is the one carried out by Munson et al. because they compare the APTIMA^®^ HPV Assay against the Cervista HPV HR, which is another commercial isothermal product based on IT. They are reporting the processing and analysis of more than 4000 samples using both tests, obtaining a concordance of 88.7%. Also noteworthy is the number of samples included in this article, giving statistical and clinical value [86]. Han et al. report the comparison between the APTIMA HPV Assay and a p16 oncoprotein immunohistochemistry in 50 samples analyzed; they declare a concordance of 92% in the results [84]. In another work, the comparison was made with the Hybrid Capture^®^ test, which is based on the detection of viral DNA. In this work, SiHa cells were used as a positive control, in addition to plasmid DNA (HPV-16) and RNA (E6/E7 mRNA), which reported a concordance of 94.2% between the tests. The LOD of the APTIMA^®^ HPV Assay mentioned is 10^3^ copies/mL (equivalent to 10^2^ copies/reaction) [85]. Finally, one of the most interesting comparisons is the one carried out by Munson et al. because they compare the APTIMA^®^ HPV Assay against the Cervista HPV HR, which is another commercial isothermal product based on IT. Reporting the processing and analysis of more than 4000 samples using both tests, obtaining a concordance of 88.7%. Also noteworthy is the number of samples included in this article, giving statistical and clinical value [86].

### 3.4. SDA

Developed in 1992 by Walker et al., it is one of the oldest INAATs. This technique is based on using a pair of primers with recognition sites for the *Hin*cII enzyme and the dNTPs: dGTP, dCTP, TTP, and thiol-modified dATP (dATPαS) (Table 3). The dATPαS nucleotide produces hemiphosphorothioate-modified DNA strands, protecting *Hin*cII restriction sites. The *Hin*cII enzyme cuts the unmodified DNA strand, leaving the modified complementary strands intact. It is used as a template by the exo-Klenow polymerase, amplifying the target sequence from the cut at the 3′ end. Also, another pair of external primers called bumper primers can be used to help the initial displacement of the target sequence [87]. The reaction is incubated at 37 °C for 1 to 5 h [88]. However, these conditions are based on the original description. Currently, with the improvement of the technology applied to enzymes and buffers, there are variants of these conditions, reducing the time and varying the incubation temperature.

A pair of research groups have used this technology to detect HPV. The first was published in 2000, and the test developed is coupled with in-situ hybridization (ISH). In this test, they used the Ca Ski cell line as a positive control for HPV-16 (500 copies/cell) for standardization. In this work, they also report the use of other sets of primers for the detection of HIV-1 and Hepatitis C [89]. The other test, which was based on SDA for the detection of HPV, was published by Yan et al. In this study, the researchers used a photothermal detection combining the use of gold nanoparticles (AuNPs), a portable laser, and a hairpin probe specific for HPV-16. In addition, they used the enzymes Nt.*Bst*NBI nicking endonuclease and *Bst* DNA polymerase. In this work, they report that 10 cervical brush samples were analyzed with a LOD of 0.3 fM [90].

### 3.5. RCA

One technique, developed in 1995 by Fire and Xu, leverages the properties of the Phi29 DNA polymerase enzyme (5′- to -3′ polymerization activity, 3′- to -5′ ssDNA exonucleolytic activity, and strong strand displacement activity) in combination with T4 DNA ligase and a primer that hybridizes to the target sequence (Table 3). This allows for DNA amplification. However, the target can also be DNA or RNA [91]. To carry out the amplification reaction, the target sequence must be circulated by ligating into padlock probes using the T4 ligase. The generated template is amplified by the Phi29 DNA polymerase using a single primer. This produces a long single strand of DNA, or various primers that can be used to amplify different regions of the circular template. This generates multiple copies and creates a highly branched sequence [87]. The amplification reaction is carried out at a temperature of 30 °C in approximately 60 min. A second study that used the same technique, and is one of the latest INAATs developed. This INAAT was developed for the detection of HPV and was published by Rao et al. This test differs in that it uses the viral RNA as a target, particularly the E6/E7 transcripts of 14 high-risk HPV genotypes (16, 18, 31, 33, 35, 39, 45, 51, 52, 56, 58, 59, 66, and 68). To achieve this, they designed a pool of padlock probes, which was exposed to the samples to achieve hybridization, ligation, and, thus, their subsequent amplification. HeLa, A549, and HepG2 cell lines were used to validate the test. This was also validated with some cervical swab samples [92].

### 3.6. IT

One of the INAATs that has not been so exploited by the scientific community for the development of diagnostic tests is called IT. This technology was developed and patented in 2002 by Third Wave Technologies (Boca Raton, FL, USA). In 2008, the company was acquired by Hologic (Marlborough, MA, USA). IT is based on amplifying a target-specific signal but not the target itself, and this is achieved using a cleavage enzyme, an upstream Invader primer, a downstream probe, and a synthetic hairpin primer with a fluorescent dye. These elements function through a mechanism where the primers and probe create an invasive structure (a one-base overlap precisely at the nucleotide). This structure is recognized by the cleavase enzyme, generating new structures and interactions that can be monitored by the fluorescence incorporated in the probes and can be used to detect either DNA or RNA. The amplification reaction is incubated at 63 °C for approximately 240 min (Table 3) [93]. Hologic currently markets two types of isothermal tests, which also have FDA approval: Cervista HPV 16/18 and Cervista HPV HR. These are tests that have been widely studied and compared by various authors [94,95,96,97]. Day et al. report the LODs of the different genotypes (16, 18, 31, 33, 35, 39, 45, 51, 52, 56, 58, 59, 66, and 68) and vary from 1250 to 5000 copies/reaction [94].

This information is also presented in the documents provided for the users of the Cervista kits. This is the most time-consuming test, requiring 4 h of incubation at 63 °C, and the results are revealed to the user using a microplate reader that analyzes fluorescence.

### 3.7. HDA

Another method was developed in 2004 by Vincent et al. The researchers aimed to amplify DNA fragments, combining a DNA helicase (UvrD), ssDNA binding protein (SSB), a DNA polymerase, and specific primers hybridize to each border of the target DNA (Table 3). The amplification reaction begins when the helicase enzyme separates the duplex DN, and the SSB proteins help stabilize the DNA strands, preventing the formation of double-stranded DNA, which allows the primers and polymerase to access the target region and start amplifying the sequence of interest. The incubation and time may vary depending on the polymerase used, originally exo-Klenow polymerase was used, with incubation at 37 °C for 60 min; other variants use the *Bst* enzyme which is incubated at 65 °C for 60 to 90 min [98].

In the literature, there is only one article based on the HDA technique which is applied to the detection of HPV. This test was designed for the detection of genotypes 16 and 18. The researchers used the IsoAmp III Universal tHDA kit (BioHelix, Beverly, MA, USA), where the reaction was incubated at 65 °C for 90 min, including primers specific to HPV-16 and HPV-18, along with the sample. For standardization, the Ca Ski and SiHa cell lines were used, and clinical samples were subsequently evaluated. Initially, DNA amplification was assessed using agarose gel electrophoresis, and later, it was performed in a real-time format by adding the EvaGreen intercalator (Biotium, San Francisco, CA, USA). The authors reported a LOD of 1 copy/reaction for HPV-16 and 10 copies/reaction for HPV-18 [99].

### 3.8. SIA

SIA is another technology that was developed in 2016 and designed for HPV detection (11, 16, and 18). This test requires a nicking endonuclease (Nt.*Bsm*AI), a Phi 29 polymerase, and a circle probe (synthesized as in RCA) (Table 3). The circle probe contains a complementary sequence and determines the target’s amplification. The reaction of this test is carried out at 37 °C for 30 min. In addition, the authors of this work performed the detection through electrochemistry using a gold electrode. As a positive control, they used the HeLa cell line [100].

### 3.9. OA

OA is a technique developed and patented in 2017 by Atila BioSystems, a company that markets various products based on OA. Some examples of these products are the iAMP COVID-19 Detection Kit (for the detection of SARS-CoV-2) and iAMP^®^ Candidiasis Detection Kit (for the detection of candida). It also markets three products for HPV detection: ScreenFire HPV RS Assay, AmpFire HPV Screening 16/18/HR, and AmpFire HPV High-Risk Genotyping. The main characteristics of OA technology are its ability to be real-time and multiplex, and that it carries out reactions in 75 min at 60 °C (Table 3). Regarding the technology’s components, it is similar to the LAMP technique since it uses a set of primers and the *Bst* polymerase, with its main difference being that the primers include artificial sequences [101]. Several articles report the performance of various OA-based tests [102,103,104]. The genotypes that are capable of detecting, taking into account all the products that Atila BioSystems handles through OA, are 16, 18, 31, 33, 35, 39, 45, 51, 52, 53, 56, 58, 59, 66, and 68. Regarding the LOD of this technique, Tang et al. report a range between 2 to 20 copies/reaction, where 214 clinical samples were evaluated [102].

### 3.10. PER

PER is one of the most recent INAATs developed. This technique primarily utilizes a polymerase (*Bst*), a fluorophore-labeled primer, and a hairpin probe, which are incubated at 37 °C for 60 min (Table 3). PER leverages the secondary structures designed in the primers and probes, which are formed in the reaction of this technique, and through fluorescence, the presence of the target can be identified [105]. Zhang et al. published in 2022 the detection of HPV-16 using the PER technique. They evaluated the test with 24 clinical cervical swab samples, reporting an LOD of 18 fM in a time of 60 min. In this work, the researchers also included designs for detecting Epstein-Barr virus, Hepatitis B virus, and *Ureaplasma urealyticum*. It is important to note that this technique is at an early stage; there are few published articles demonstrating its use. Therefore, it requires further maturation and validation [106].

### 3.11. MCLSA

A more recently proposed INAAT is the MCLSA, which was published in 2021. This new technique was designed for the detection of HPV-16 and is based on the use of the *Bst* polymerase and the design of a set of 5 primers, which target seven distinct regions in the viral genome (E6/E7 genes). This technique is carried out at 62 °C for 45 min (Table 3). The most interesting feature of this test is that it includes SYBR Green to reveal the amplification of the target in a simple way. This test was evaluated in 46 patient samples, reporting a LOD of 54 copies/reaction [107]. MCLSA is in a similar developmental stage as PER, having recently emerged. It requires additional study and application across diverse research groups.

**Table 3:** Main characteristics of INAATs.

Technique	Enzymes Required	# Primers	Temperature	Time (min)	Developed	Commercial (HPV)	Pros	Cons
NASBA	AMV T7 RNA polymerase RNase H	2	41 °C	90–120	1991	No	Ideal for detecting RNA targets	Not optimal for DNA targets Reaction time
SDA	*Hin*cII exo^−^ Klenow polymerase	2–4	37 °C	120	1992	No	Temperature	Primer design Reaction duration Inefficiency with long sequence amplification
RCA	Phi29 DNA polymerase T4 DNA ligase	1	30 °C	60–90	1995	No	Temperature	Works with circular templates Inefficiency with long sequence amplification
TMA	M-MLV T7 RNA polymerase	2	41 °C	90–120	1995	Yes	Ideal for detecting RNA targets	Not optimal for DNA targets Reaction time
LAMP	*Bst*	4 or 6	60–65 °C	<60	2000	Yes	Availability of commercial kits Products with colorimetric detection	Primer design False positives
IT	Cleavase	3	63 °C	240	2002	Yes	SNP detection	Reaction time
HDA	DNA Helicase SSB *Bst*	2	65 °C	90	2004	No	Primer design Availability of commercial kits	Reaction time
RPA	*Bsu* gp32 uxsX uvsY	2	37–42 °C	20–40	2006	Yes	Primer design Temperature Reaction time	Number of required enzymes Commercial access
SIA	Nt.*Bsm*AI Phi29 DNA polymerase	1	37 °C	30	2016	No	Temperature Reaction time	Commercial access Experimental Stage
OA	*Bst*	6	60 °C	<60	2017	Yes	Reaction time	Primer design
PER	*Bst*	2	37 °C	60	2017	No	Temperature	Commercial access Experimental Stage
MCLSA	*Bst*	5	62 °C	45	2021	No	Reaction time	Commercial access Experimental Stage

NASBA (Nucleic acid sequence-based amplification), SDA (Strand-displacement amplification), RCA (rolling circle amplification), TMA (transcription-mediated amplification), LAMP (loop-mediated isothermal amplification), IT (Invader Technology), HDA (helicase-dependent amplification), RPA (Recombinase polymerase amplification), SIA (self-primed isothermal amplification), OA (Omega Amplification), PER (Primer exchange amplification, MCLSA (multiple cross-linking spiral amplification).

## 4. Commercial and FDA-Approved Molecular Test for HPV Detection

A pivotal aspect of the detection of HPV lies in the commercialization of the developed molecular tests. This part is crucial for effectively translating advancements into the implementation in the field, ultimately facilitating the achievement of 70% screening coverage (strategy 90-70-90) [23].

Therefore, various companies worldwide have been marketing various assays for HPV detection for decades. These assays cover a wide range of use of technologies ranging from traditional methods, such as the use of PCR accompanied by electrophoresis to newer ones based on LAMP. Recently, a comprehensive analysis identified at least 264 commercial HPV detection tests globally. Notably, most of these assays do not have reliable clinical or published research support, and more than 79% do not have a regulatory evaluation [108].

In many countries, to be able to use tests for diagnostic purposes in people (being the primary objective), it is necessary to receive approval from a health authority. In the U.S.A., the FDA oversees the regulation of these tests, and currently, there are only 9 tests that have been approved to be used to detect HPV [108].

The first FDA-approved test was the Digene Hybrid Capture 2 High-Risk HPV DNA Test in 2003. Currently, Qiagen is marketing this product. Among the tests approved by the FDA, which are based on the detection of viral DNA using qPCR, there are also isothermal methods based on TMA and Invader technology. Most of these tests can detect the DNA of the primary high-risk HPV genotypes (16, 18, 31, 33, 35, 39, 45, 51, 52, 56, 58, 59, 68) [108]. However, only the two tests marketed by Gen-Probe, Inc. are designed to detect viral RNA. Table 4 shows the list of tests approved by the FDA and the main features.

## 5. Challenges and Perspectives

INAATs have multiple advantages over the qPCR for HPV detection since they do not require sophisticated laboratories or equipment and highly qualified personnel. Additionally, results can be viewed in less than an hour. Likewise, INAATs are highly sensitive and allow large-scale samples to be analyzed [21,22]. But despite their multiple advantages, INAATs present a series of limitations such as primer design (depends on the INAATs to be used), transportation of reagents, production costs, and sample processing, among others, that do not allow them to be used globally [109,110].

Regarding the design of primers, some isothermal tests such as LAMP or SDA require complex design primers, which can alter the results if the primers present complementarity between them or form strong secondary structures at the temperatures at which target amplification occurs [109,110]. Therefore, it is necessary to use software that allows the design of primers depending on the isothermal test to be used; this will help reduce the probability of the formation of secondary structures in the primers or the complementarity between them. In addition, it will make the standardization process more efficient.

On the other hand, the transport of reagents is another critical point because the enzymes (polymerases, recombinases, helicases, ligases, among others) can lose their activity if adequate cold chain logistics is not maintained. Although lyophilized master mixes containing all the essential components for the amplification reaction are currently used (to avoid this type of problem), a significant limitation is that they are not available for all INAATs or all countries [111,112]. Furthermore, it is essential to highlight that the production costs of INAATs can vary significantly depending on the region. In some countries, the need to import reagents can increase the overall production and sales costs [22,112,113]. In addition, there is the possibility that the reagents are lost along the way (due to depending on multiple intermediaries) or that their arrival takes longer than estimated due to being detained in the countries’ customs [113]. A clear example that represents these limitations is the TwistAmp^®^ exo product, which is sold by the company TwistDx Ltd. located in Cambridge, UK [114]. This product uses the RPA principle and, for its import, requires a temperature of −20 °C. Although it can tolerate room temperature for days without losing its activity, it costs around 372 GBP, plus shipping costs, making its production more expensive in other countries. In this sense, it is necessary for companies that manufacture the reagents for the isothermal tests are located in different strategic countries, since this will allow the reduction of costs and shipping times. This would promote making HPV screening using INAATs available to the entire population and that it can be implemented quickly.

Another point of great importance is the processing of the sample, which must be compatible with the INAAT to be carried out to avoid false negatives because some platforms, such as SDA, RCA, or NASBA, among others, are sensitive to contaminating agents coming from the sample that can act as inhibitors of the amplification reaction [109,110]. Therefore, it is necessary to generate kits or platforms combined with INAATs that eliminate all possible contaminants to ensure the precision of the results.

Also, it is essential to mention that some of the detection methods used in INAATs present disadvantages, such as doubling the time to view results, not being specific to the target, or increasing the test costs [109,110]. For example, the electrophoresis technique doubles the time to identify the presence of the pathogen of interest and requires a laboratory [28]. The dyes are considered non-specific since they depend on the intermediates generated during the amplification reaction, such as pH changes or magnesium pyrophosphate precipitation, to determine the existence of HPV [30,31,32,33,34,35,36]. Likewise, fluorophores have been used, which are more expensive and can generate false positives (if the primers are non-specific) since it is intercalated in any double-stranded product [37,38,39,40,41,42]. To provide more precise detection methods, probes have been used, which require incubation after the amplification reaction, which increases the time needed to observe the results [48]. Also, INAATs have been accompanied by platforms such as microfluidics, CRISPR-Cas, LFA, and biosensors, which sometimes require more than 60 min to determine the presence of the target HPV genotype [22]. In this sense, more specific and sensitive methods that are compatible with INAATs and that do not require more time after the amplification reaction are needed to visualize the results.

Although some INAATS have been developed for a long time, to date, there are only two commercial products approved by the FDA. Among some of the reasons for its poor incorporation into the market is that some tests require complex primer designs or are protected by patent. This could be solved by developing commercial primers for the different INAAT tests that guarantee amplification of the target HPV genotype. Concerning patented INAATs, the licensing of patents can be generated to authorize companies to develop and produce the INAATs of interest.

Furthermore, despite the INAATs meeting the ASSURED criteria and that devices connected to the smartphone have been developed, it is still necessary to make more efforts so that depending on the result (if positive), these can be transmitted to clinics or medical centers to advise the patient; and thus, comply with the Real-time connectivity criterion, forming the new acronym REASSURED [24].

## 6. Conclusions

INAATs are an up-and-coming technology since they present multiple advantages over the qPCR for the molecular diagnosis of HPV, as they do not require sophisticated equipment, highly trained personnel, and specialized laboratories. Furthermore, the working temperature is constant (20 °C to 65 °C), and its LODs range from one copy to hundreds of thousands of copies (like qPCR). Usually, the primary detection methods used are colorimetric and fluorescence, allowing to see results in less than an hour, but since they are considered non-specific, the use of probes has been chosen to increase sensitivity and specificity, requiring incubations after amplification and taking more time for the results. In this sense, to increase sensitivity, the INAATs have been accompanied by technologies such as CRISPR-Cas, microfluidics, LFA, or biosensors, and the results can take from one hour up to two hours since some of the techniques require prior incubation after the amplification reaction. Despite these technologies allowing the REASSURED criterion to be met, it is still necessary to develop platforms that enable contact with clinical centers and health specialists to advise patients in case of positive results.

Despite the extensive research carried out for a long time and the important technological developments by various research groups, only two commercial tests for HPV detection have received FDA approval, and their low implementation is because some INAATs require multiple enzymes, primers with complex designs, reagents that are not available for all countries, and some of the INAATs are patented.

Therefore, it is vital to develop dissemination and marketing strategies through local or regional suppliers that sell consumables for INAATs and allow production costs to be reduced. Furthermore, this will help promote its large-scale incorporation into different health systems worldwide; and be able to perform routine INAATs, which offer timely results to prevent the development of various types of cancer related to HPV (cervix, penis, oropharynx, vagina, vulva, and anus) and provide timely treatment, mainly in rural areas or in precarious conditions.

## Figures and Tables

**Table 4:** List of FDA Approved Molecular Tests for HPV detection with the main features.

Product	Technology	Company	Molecule (Target)	Gene (Target)	Genotype (Detection)	Year Approved
Digene Hybrid Capture 2 High-Risk HPV DNA Test	Hybrid capture technology	Digene Corporation (Qiagen)	DNA	-	16,18,31,33,35,39,45,51, 52,56,58,59, 68	2003
Cervista HPV 16/18	Invader Technology	Hologic, Inc.	DNA	L1	16,18	2009
Cervista HPV HR	Invader Technology	Hologic, Inc.	DNA	L1	16, 18, 31, 33, 35, 39, 45, 51, 52, 56, 58, 59, 66, 68	2009
COBAS HPV Test	PCR	Roche Molecular Systems, Inc.	DNA	L1	16, 18, 31, 33, 35, 39, 45, 51, 52, 56, 58, 59, 66, 68	2011
APTIMA HPV Assay	TMA	Gen-Probe, Inc.	RNA	E6/E7	16, 18, 31, 33, 35, 39, 45, 51, 52, 56, 58, 59, 66, 68	2011
APTIMA HPV 16 18/45 Genotype Assay	TMA	Gen-Probe, Inc.	RNA	E6/E7	16, 18, 45	2012
BD ONCLARITY HPV ASSAY	PCR	Becton, Dickinson, and company	DNA	E6/E7	16, 18, 31, 33, 35, 39, 45, 51, 52, 56, 58, 59, 66, 68	2018
Cobas HPV for use on the Cobas 6800/8800 Systems	PCR	Roche Molecular Systems, Inc.	DNA	L1	16, 18, 31, 33, 35, 39, 45, 51, 52, 56, 58, 59, 66, 68	2020
Alinity m HR HPV	PCR	Abbott Molecular, Inc.	DNA	L1	16, 18, 31, 33, 35, 39, 45, 51, 52, 56, 58, 59, 66, 68	2023

Isothermal tests are shown in red.

## Data Availability

No new data were created or analyzed in this study. Data sharing is not applicable to this article.
